# Fertility histories and chronic conditions later in life in Europe

**DOI:** 10.1007/s10433-018-0494-z

**Published:** 2018-11-29

**Authors:** Maria Sironi

**Affiliations:** Department of Social Science, University College London, 20 Bedford Way, London, WC1H 0AL UK

**Keywords:** Fertility, Health, Ageing, Europe, SHARE, Chronic diseases

## Abstract

Understanding the association between fertility histories and health later in life is necessary in the context of ageing societies. Past literature has generally found a U-shaped relationship between parity, age at first birth, and several health-related outcomes. However, these findings differed to some extent depending on the country under analysis and on the measures of health considered. As such, using wave 3 (2008–2009) and 5 (2013) of the Survey of Health, Ageing and Retirement in Europe (SHARE), this work aimed to answer the question: “Are fertility histories associated with the presence of chronic conditions later in life in Europe?” The analysis included 11 European countries and compared results using two different measures of chronic conditions: self-reported chronic or long-term illness and chronic diseases diagnosed by a doctor. Results showed that age at first birth is more relevant than parity for health outcomes at older ages. Moreover, in socio-democratic and continental countries, the association between fertility and chronic conditions—in particular between age at first birth and long-term illnesses—is statistically significant among women, but not among men. Finally, the association between fertility history and health was similar when using self-reported measures and chronic diseases diagnosed by a doctor.

## Introduction

It is well known that life expectancy in developed countries has increased for decades, and this pattern is likely to continue thanks to medical innovations. Over the same period of time, fertility rates have decreased substantially. Given the decrease in mortality rates and fertility rates, the age structure of industrialized societies has changed and shifted towards an older population (European Commission 2014), and health inequalities among older people have become starker (Marmot 2005). An overview of health conditions among older adults in Europe showed that inequalities in self-reported health and disability persist in old age (Huisman et al. 2003). Consequently, heightened morbidity and longer periods spent with a lower quality of life have become serious threats for larger segments of the population. Chronic diseases—in particular cancer and diseases of the circulatory system—are the main cause of death and of disability in European countries, especially among older adults (Eurostat 2016). The rise in chronic conditions is reflected in longer life spans with the disease, due to earlier and more accurate diagnosis (Kuh and Shlomo 2004; Kuh et al. 2013). Hence, it is important to focus on this outcome and identify major risk factors to better understand the ageing process.

Several life course theories hypothesize that health in adulthood is the result of early life conditions and even critical moments in utero or early infancy (Barker 1997, 2004; Roseboom et al. 2001; Barker et al. 2002) and that health among older people is driven by a continuous and cumulative process that develops over the life course (Halfon and Hochstein 2002). According to these models, health at a specific point in time is determined by all of the events that happened previously and the dynamic interaction between them. Therefore, it is important to take into account key events across the life course to fully understand health conditions among older people (Kuh and Shlomo 2004). Among these events and because of the decline in fertility rates, childbearing has attracted a lot of attention in the demographic, epidemiological, and ageing literatures. The consequences of fertility histories on mortality and health have been widely investigated. Past research has shown the existence of a relationship between parity and age at first birth and mortality (Doblhammer 2000; Grundy and Kravdal 2007, 2010; Grundy and Tomassini 2006; Hinkula et al. 2005; Hurt et al. 2006; Jaffe et al. 2009, 2011; Tamakoshi et al. 2010). More recently, studies have also found an association between fertility and physical and mental health outcomes in middle and old age, such as self-rated health, chronic conditions, and depression (Buber and Engelhardt 2008; Grundy and Tomassini 2005; Grundy and Foverskov 2016; Grundy and Holt 2000; Gunes 2016; Hank 2010; Hanson et al. 2015; Henretta 2007; O’ Flaherty et al. 2016; Pirkle et al. 2014; Read et al. 2011; Williams et al. 2011).

However, most of the previous studies have either focused on a single country or on the comparison between two countries or small groups of countries. Therefore, it is necessary to extend cross-national comparisons given all of the factors (discussed below) that can make the association between fertility and health different in various contexts. Existing literature has primarily used self-reported measures of health and has not compared these associations across different measures of the same health conditions. Thus, to overcome these gaps in the literature and to understand the determinants of ageing patterns of older adults, this study included several European countries and investigated the role of fertility histories in shaping health later in life. In particular, I analysed chronic conditions, using both strictly self-reported measures of long-term illness and self-reported doctor diagnoses.

## Literature review and theoretical background

As previous studies have shown, there are several mechanisms through which fertility can affect health and mortality (Goisis and Sigle-Rushton 2014; Grundy and Tomassini 2005). The primary direct mechanism is the effect of pregnancy on a woman’s body. It can cause immediate disadvantages, such as post-partum depression and weight gain, and other consequences due to physiological stress. However, childbirth can also cause a rise in a woman’s stress resilience and it can benefit medical care and social integration. The mother’s age, her marital and employment status, the number of children already born, and other socio-economic characteristics affect these consequences. Breastfeeding has been found to have an impact on maternal health (Heinig and Dewey 1997), such as preventing incidence of diabetes, heart disease, and breast cancer. There are also indirect mechanisms by which fertility can affect health, like the social status associated with being a parent, the social support, and the intergenerational transfers implied by parenthood. These social mechanisms affect both men and women and can have both positive (e.g. social support) and negative effects (e.g. economic strain, stress). In particular, the negative effects are likely to be stronger for young parents (e.g. teenage parents), who have less resources to cope with stress and financial difficulties (Falci et al. 2010). The implications of these social mechanisms are likely to persist in older age, when social support and social networks are key factors for healthy ageing.

Previous literature has found that the most consistent result is a U-shaped relationship between parity and mortality/health, with individuals having two or three children having the lowest mortality risk and best health outcomes (Grundy 2009; Kravdal et al. 2012; Spence and Eberstein 2009). Teenage childbearing, short birth intervals and the death of a child have negative consequences on health (Grundy and Read 2015; Hank 2010; Henretta 2007; Read et al. 2011). Late age at childbearing is associated with better physical health but worse mental health later in life (Spence 2008; Read and Grundy 2011). All these associations are partially mediated by marital history, socio-economic factors, and health behaviours.

However, results differ to some extent depending on the country and on the measures of health that are considered. The negative impact of childlessness, high parities, and early age at first birth seems to be stronger for women in Great Britain and Germany (Grundy and Tomassini 2005, 2006; Hank 2010), while it is stronger among men in Egypt and Australia (Engelman et al. 2010; O’ Flaherty et al. 2016). The relationship between fertility and health is mitigated by socio-economic factors in the USA, yet that is not the case in the UK (Henretta et al. 2008).

The fact that fertility histories affect the health of men and women in different ways across varying contexts and that socio-economic and lifestyle variables play a more important role in some locations than in others suggests that the mechanisms and the channels through which this association between fertility and health works are not universal. There are several aspects to consider that might contribute to cross-national variations. Firstly, historical contexts might influence individual fertility behaviours and create differences across countries. For example, the Great Depression hit some countries harder than others (e.g. Italy and the Netherlands more than Spain) and later than others (e.g. France). Thus, it is necessary to consider the long-term effects of the event on affected economies and on demographic behaviours, including fertility. Another example is World War II and the resulting Baby Boom in particular countries, such as France and Germany. Secondly, fertility-related welfare transfers, such as childcare provisions and gender equality policies, can mitigate the economic burden and stress associated with childbearing, especially for vulnerable groups (e.g. young parents or large families). Consequently, in Northern Europe, where welfare provisions are generous, it is plausible to expect less negative associations between fertility events and health later in life. Thirdly, cultural aspects such as gender roles and gender norms could explain cross-national gender differences in the association between fertility history and health. The contrasts between gender equality at the institutional level (e.g. education and labour market) and at the individual level (e.g. division of housework) have been considered as key factors for fertility decisions (McDonald 2000a, b, 2006; Esping-Andersen and Billari 2015), and it can impact the way in which fertility trajectories affect health. Hence, Southern and Eastern European countries, with low levels of gender equality, might show lower levels of fertility and stronger associations between fertility and chronic conditions among women than among men. Additionally, there are variations in the national health care systems, and this can influence the way in which individuals access and utilize health services. Finally, the selection into specific fertility pathways (e.g. early first birth or high parity) might vary across countries.

Moreover, previous studies differ in the way in which health is measured, and this may be the reason why findings are not always consistent. Recent literature using objective measures (biomarkers in particular) (Grundy and Read 2015; Hardy et al. 2007; Lacey et al. 2016) has shown that number of children is not significantly associated with health later in life, or that the association disappears after controlling for health behaviours and lifestyle. Hence, it is relevant to compare different measures of the same health conditions to have a more comprehensive picture. It is important to note that the variation in fertility rates across Europe has decreased over time. Therefore, among younger cohorts, the association between parity and health might be less strong than in the past or less clear-cut than in societies with higher levels of fertility.

Using wave 3 (2008–2009) and 5 (2013) of the Survey of Health, Ageing and Retirement in Europe (SHARE), this study aimed to answer the question, “Are fertility histories associated with the presence of chronic conditions later in life in Europe?” Furthermore, I looked at differences in this association across 11 European countries and compared results using two different measures of chronic conditions.

## Data and methods

SHARE is a cross-national panel data study, which started in 2004 and involved interviews with individuals aged 50 and above in 21 European countries. SHARE is the first survey in Europe to focus specifically on ageing and to look at health conditions of a growing group of the population. For the purpose of this analysis, only wave 3 (2008–2009) and wave 5 (2013) were used. Wave 3, also known as SHARELIFE, focuses on people’s life histories and contains information on several areas, such as fertility and partnership histories, housing and work histories, childhood health, and early life conditions. Wave 5 includes important variables on individuals’ health. The target population^1^ of individuals in wave 5 is defined as “persons born in 1962 or earlier, and persons who are a spouse/partner of a person born in 1962 or earlier” (Malter and Börsch-Supan 2015). The survey sample design is different across countries (Börsch-Supan and Jürges 2005), and the response and retention rates vary by wave and country^2^ (Börsch-Supan 2018).

The analyses were restricted to respondents who were interviewed both in wave 3 and in wave 5, and who were 50 years old or older in 2013 (15,116). Twenty-five individuals were excluded because they did not answer the chronic conditions questions in wave 5, and 382 were excluded because they did not have information on the other variables included in the analysis. The final sample consisted of 8289 women and 6420 men, from 11 European countries: Austria, Germany, Sweden, the Netherlands, Spain, Italy, France, Denmark, Switzerland, Belgium, and Czech Republic.^3^

### Measures of health

Three different dependent variables from wave 5 were used in this analysis regarding chronic conditions. The first variable was *self*-*reported long*-*term illness*. Respondents were asked the question, “Some people suffer from chronic or long-term health problems. By chronic or long-term, we mean it has troubled you over a period of time or is likely to affect you over a period of time. Do you have any such health problems, illness, disability or infirmity?” (yes or no). The other two variables were *measures of chronic conditions as diagnosed by a doctor*. Individuals in the sample were shown a card with a list of conditions,^4^ and they answered the question, “Has a doctor ever told you that you had/Do you currently have any of the conditions on this card? Please tell me the number or numbers of the conditions.” The answer to this question was used to build (a) a dichotomous variable equal to 1 if the respondent was diagnosed with at least one chronic condition, and 0 otherwise, and (b) a variable corresponding to the number of diagnosed chronic conditions. Although these two variables are not strictly objective measures, as the respondent reports them they provide a more comprehensive picture together with the *self*-*reported long*-*term illness* variable, given that they imply a doctor diagnosis (and so access and active use of health care services).

### Measures of fertility

Several independent variables representing the fertility trajectory were used in the analysis: number of children, age at first birth and age at last birth, short birth interval (equal to 1 if the distance between two births is less than 2 years), long birth interval (equal to 1 if the distance between two births is more than 5 years), and experiencing the death of a child (equal to 1 if the responded reported the death of a child). The use of several variables on fertility offered a more detailed picture than just using parity, or age at first birth, and could help identify other aspects of childbearing that can be important risk factors for later health.

### Control variables

The control variables included in the analysis were *age at interview* at wave 5, level of *education*,^5^ if *retired* from work at wave 5, *partnership status*,^6^ the *number of marriages*, if *ever cohabited* with a partner, *early life conditions*,^7^ and *childhood health*^8^ included in wave 3. Control variables were identified through the past literature and found to be associated with health and/or fertility histories. For example, childhood health (Barker 1997; Case et al. 2005) and early life conditions (Campbell et al. 2014; Cohen et al. 2010; Duncan et al. 2010) have shown a strong correlation with adult health. Moreover, education, occupation, and partnership histories could influence the fertility trajectory, given that the number of children and the timing of childbirth vary with different levels of SES (Clark and Cummins 2009; Clark and Hamilton 2006; Dribe et al. 2014; Skirbekk 2008) and are mediated by the presence of a partner (O’Leary et al. 2010).

### Statistical analysis

Because of the previous findings in the literature showing a differing impact of childbearing on men and women, all of the analyses were performed separately by gender. Following some descriptive statistics^9^ of the variables used in the analysis by gender and by country, multivariate regression models were run to estimate the association between the different fertility variables and chronic conditions.^10^

First, to have a general picture of the associations in Europe, an analysis was performed on all the countries together. Self-reported long-term illness was looked at by running six logistic models, each one focusing on a different fertility characteristic: parity, age at first and last birth, short and long birth interval, and death of a child. Each specification included the control variables, the number of children, and country dummies. Then, the *presence* of diagnosed chronic diseases (also in this case using six logistic regression models) was looked at, and at the *number* of chronic diseases using negative binomial regression models.

Secondly, the models were run for *self*-*reported long*-*term illness* and for the *presence* of diagnosed chronic diseases by country. Comparing the results across different measures of chronic conditions allowed an evaluation of whether the results were influenced by the type of measure used. The analyses were performed using STATA, reporting different levels of statistical significance of the coefficients (*p < 0.10, **p < 0.05, ***p < 0.01).

## Results

### Descriptive results

Tables 1 and 2 show heterogeneity in the prevalence of chronic conditions across European countries. The prevalence of self-reported long-term illnesses was lowest in Switzerland (35.2% for women; 33.1% for men) and Italy (48.6% for women; 42.5% for men) and highest in Germany (64.8% for women; 66.9% for men) and Spain (66.2% for women; 58% for men). This heterogeneity was reflected also when looking at diagnosed chronic diseases. In Switzerland, there was the lowest prevalence of chronic diseases for both men and women (e.g. 67.5% and 1.2 conditions on average among women). The highest prevalence and number of chronic diseases were observed in Spain and Czech Republic for women and in Germany and Czech Republic for men.

**Table 1:** Sociodemographic characteristics and health—descriptive statistics—women

Sociodemographic characteristics	AT	DE	SE	NL	ES	IT	FR	DK	CH	BE	CZ	ALL
Age at interview	69	73	70	73	71	72	72	71	70	70	72	69
Education (%)												
No education	0	1	0	1	19	5	15	0	0	1	0	8
Primary, lower secondary	42	18	42	56	67	73	35	25	39	49	51	49
Upper secondary	43	55	31	23	9	19	30	34	54	26	43	28
Tertiary	15	26	27	20	5	4	21	42	7	23	7	15
% Retired	73	67	77	54	31	53	71	65	56	56	86	58
Partnership status (%)												
Single	8	4	8	5	8	5	8	6	10	4	1	6
Married or cohabiting	47	63	61	63	66	67	56	57	50	65	56	62
Divorced	10	11	13	10	3	1	13	15	16	8	10	8
Widow	35	22	18	22	23	27	24	22	25	23	33	24
Number of marriages (%)												
Never	7	4	9	8	8	5	8	6	11	4	1	6
1 Marriage	85	82	78	84	91	93	84	76	82	87	89	87
2+ Marriages	8	14	13	8	1	2	8	17	7	9	10	7
% Ever cohabited	8	9	19	6	2	2	10	20	12	7	5	7
% Ever had a child	91	91	91	89	90	91	91	92	84	90	98	91
Number of children	2	2	2	2	3	2	2	2	2	2	2	2
Age at first child^a^	24	24	25	26	25	25	25	24	26	25	23	25
Age at last child^a^	30	30	30	30	33	31	30	30	30	30	27	31
% Short birth interval (< 2 years)^b^	28	26	15	29	24	18	27	18	31	30	18	24
% Long birth interval (> 5 years)^b^	33	34	29	18	39	35	32	30	19	22	22	33
% Death of a child^a^	12	11	8	7	14	7	9	8	10	12	8	10
Early life conditions												
% Parents with high education^d^	52	81	25	20	6	5	25	58	62	23	72	29
% Living with both parents^c^	75	79	84	90	88	91	85	88	91	90	88	86
# People per bedroom^c^	2	2	2	2	2	3	2	1	1	1	3	2
% Enough books for a bookshelf^c^	27	45	60	45	18	11	34	58	53	34	62	30
Housing^c^												
% Having a fixed bath	26	42	50	14	22	20	25	48	51	27	65	28
% Having cold running water	56	81	81	89	50	49	73	85	91	63	81	66
% Having hot running water	26	29	56	46	16	19	42	49	62	29	17	30
% Having inside toilet	42	54	60	81	40	43	47	66	74	39	69	49
% Having central heating	6	15	55	6	4	7	22	45	36	13	11	14
% Health < “very good”	35	51	27	49	36	29	40	25	44	31	21	38
% Had vaccines in childhood	95	99	97	86	90	95	96	99	93	95	97	95
% With health care source	91	90	90	97	97	95	93	99	96	97	98	94
% Missing school for 1+ month	19	21	12	23	8	7	16	12	16	18	21	14
% In hospital for 1+ month	8	11	8	8	2	3	4	7	8	5	8	5
Adult health												
% Long-term illness	57	65	58	58	66	50	49	55	35	54	56	56
% 1+ chronic diseases	81	83	79	77	88	85	83	80	68	83	85	84
# Chronic diseases	2	2	2	2	3	2	2	2	1	2	2	2
*N*	363	545	716	829	863	991	860	858	519	1087	658	8289

^a^Had at least one child

^b^Had 2+ children

^c^At age 10

^d^≥ Upper secondary. Weighted sample

**Table 2:** Sociodemographic characteristics and health—descriptive statistics—men

Sociodemographic characteristics	AT	DE	SE	NL	ES	IT	FR	DK	CH	BE	CZ	ALL
Age at interview	71	70	71	69	71	71	70	69	69	70	69	70
Education (%)												
No education	0	0	0	0	16	1	9	0	0	1	0	5
Primary, lower secondary	17	6	45	38	66	70	25	14	22	41	48	41
Upper secondary	53	57	30	30	8	22	39	47	64	28	34	32
Tertiary	29	37	25	31	10	7	27	39	14	30	18	21
% Retired	90	75	69	68	74	83	78	59	56	81	78	77
Partnership status (%)												
Single	7	9	8	7	12	7	7	10	6	5	3	8
Married or cohabiting	79	76	75	80	79	83	77	70	77	82	85	79
Divorced	6	6	10	7	4	3	8	12	11	5	5	6
Widow	7	8	7	6	5	6	8	8	6	8	8	7
Number of marriages (%)												
Never	7	9	12	11	13	8	7	9	8	5	2	9
1 Marriage	83	78	73	80	84	88	80	75	78	87	85	83
2+ Marriages	11	12	16	9	3	4	13	16	14	8	13	9
% Ever cohabited	8	9	24	7	3	5	12	22	17	8	3	8
% Ever had a child	87	84	88	84	84	88	90	86	84	89	96	87
Number of children	2	2	2	2	2	2	2	2	2	2	2	2
Age at first child^a^	27	28	28	29	28	29	27	27	29	27	26	28
Age at last child^a^	33	32	33	33	35	35	33	32	34	32	30	34
% Short birth interval (< 2 years)^b^	28	21	15	28	25	16	22	15	26	28	15	21
% Long birth interval (> 5 years)^b^	37	31	30	15	40	37	32	29	20	19	22	32
% Death of a child^a^	6	5	6	7	11	7	6	5	6	9	4	7
Early life conditions												
% Parents with high education^d^	62	85	24	21	4	6	26	60	64	21	73	30
% Living with both parents^c^	82	75	83	89	89	91	86	89	88	90	91	86
# People per bedroom^c^	2	2	2	2	2	3	2	1	1	1	2	2
% Enough books for a bookshelf^c^	29	46	61	45	18	10	35	58	50	30	58	30
Housing^c^												
% Having a fixed bath	27	36	62	14	21	19	29	53	57	22	68	28
% Having cold running water	58	84	86	92	52	48	76	87	92	68	80	68
% Having hot running water	23	26	66	50	19	17	45	55	65	27	21	31
% Having inside toilet	40	54	68	82	40	40	50	70	75	41	71	50
% Having central heating	7	15	63	8	4	5	23	49	44	11	14	15
% Health < “very good”	32	50	23	45	36	24	38	20	32	28	21	35
% Had vaccines in childhood	97	96	97	90	84	95	98	99	94	97	97	94
% With health care source	91	86	90	98	95	94	92	100	96	98	99	93
% Missing school for 1+ month	18	20	12	17	9	7	16	12	17	16	20	13
% In hospital for 1 + month	11	10	8	9	2	3	6	10	7	4	7	6
Adult health												
% Long-term illness	53	67	48	48	58	43	49	50	33	47	54	52
% 1+ chronic diseases	79	85	73	70	85	79	81	74	65	76	87	80
# Chronic diseases	2	2	1	1	2	2	2	2	1	2	2	2
*N*	238	482	575	632	693	803	633	700	386	852	426	6420

^a^Had at least one child

^b^Had 2+ children

^c^At age 10

^d^≥ Upper secondary. Weighted sample

More than 82% of the sample had been married at least once, and more than 87% had at least one child. Women had on average 2.4 children, while men had 2.2 children. The level of childlessness was highest in Switzerland and the Netherlands and lowest in Czech Republic among women, while it was more homogeneous among men. The mean age at first birth ranged between 23.2 (Czech Republic) and 25.9 years (Switzerland) for women, and between 25.8 (Belgium) and 29.3 years (Switzerland) among men. The highest age at last birth was reported in Spain (32.6 and 35.1 years for women and men, respectively), while the lowest in Czech Republic (27.4 and 30.0 years for women and men, respectively). Just below one-fourth (23.7%) of women and 21% of men experienced short birth intervals, 32.5% of women and 32% of men experienced long birth intervals, and 9.7% of women and 7.1% of men experienced the death of a child.

Tables 1 and 2 show a great level of heterogeneity in early life conditions and childhood health. For example, 5% of the respondents in Italy and Spain had parents with a high level of education, compared to 80% of the respondents in Germany. The proportion of those who missed school or that were hospitalized for more than a month when 10 years old was lowest in Italy and Spain and highest in Germany, the Netherlands, and Czech Republic.

### Multivariate results (pooled sample)

**Fig. 1:**
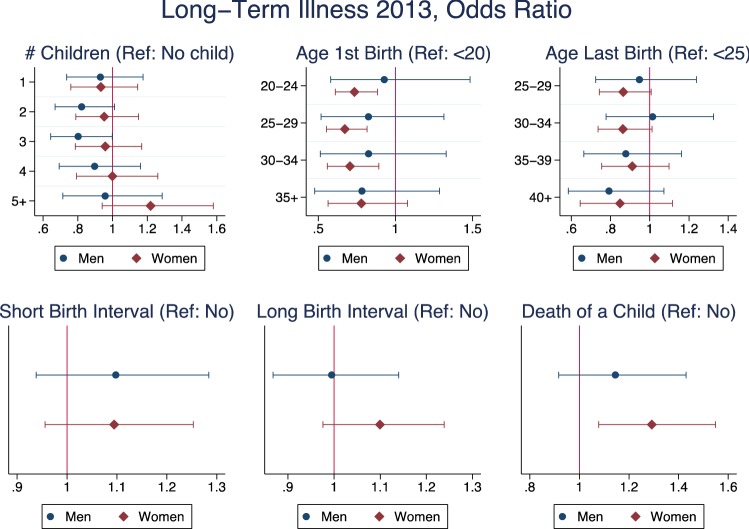
Logistic regression—long-term illness

The results for the multivariate regressions on self-reported long-term illness are reported in Fig. 1. The number of children was not relevant for the presence of long-term illnesses among women, and among men, a J-shaped relationship was observed, but the odds ratios were not significantly different from zero. Age at first birth was important among women, since those who had their first child after age 20 and before age 35 had a lower probability of reporting long-term illnesses. Age at last birth and short and long birth intervals did not have any significant association with the probability of having a long-term illness. Experiencing the death of a child was associated with a higher risk of long-term chronic conditions only among women.

**Fig. 2:**
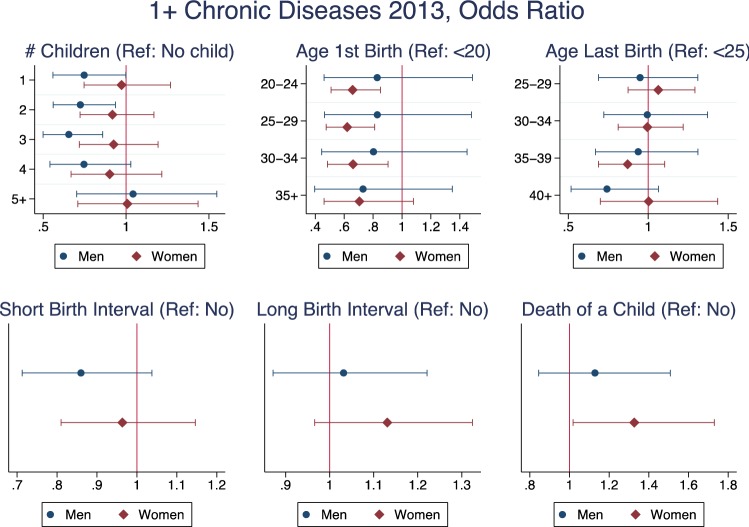
Logistic regression—chronic disease

When looking at the prevalence of diagnosed chronic diseases (Fig. 2), the results were very similar to those found on self-reported illness, except for one notable difference. The number of children was significant for men—i.e. childless men had a higher probability of reporting chronic diseases than men with children, and the odds ratios for those with one, two, three, or four children were very similar to each other, even though only the odds ratios for men with two and three children were statistically significant.

**Fig. 3:**
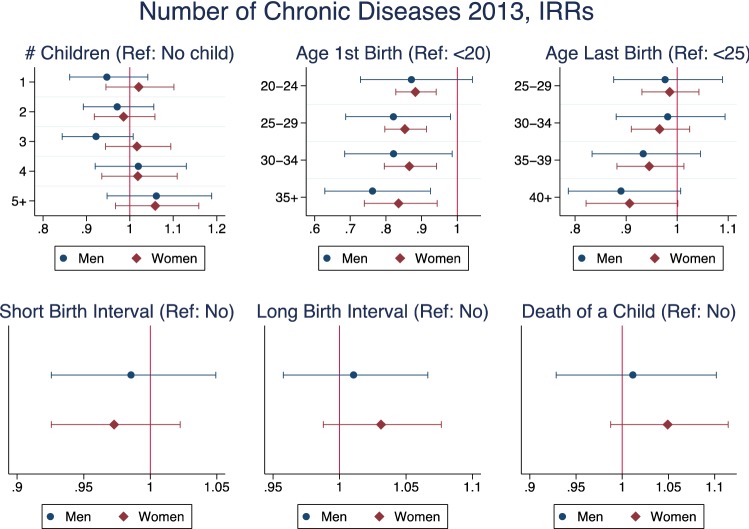
Negative binomial regression—number of chronic diseases

The only significant result for the model studying number of chronic diseases (Fig. 3) was that the prevalence rate for women who had their first child after age 20 was lower than for those who experienced a teenage pregnancy. The same was true for men who had their first child after age 25.

### Multivariate results (by country)

Tables 3 and 4 report the results by country for models studying self-reported long-term illnesses, for women and men, respectively, while Tables 5 and 6 report the results for diagnosed chronic diseases. There was a higher probability of having a long-term illness among women who had 5 or more children in Austria and France (OR 5.59 and OR 2.73, *p* value < 0.05, respectively), while the results were not significant for men. These associations were not significant in Southern and Eastern European countries. The results for age at first birth showed a U-shaped relationship between age at first birth and the risk of reporting a long-term illness among women in Sweden (OR, age 20–24 = 0.52, OR, age 25–29 = 0.467, OR, age 30–34 = 0.455, *p* value < 0.05) and Denmark (OR, age 25–29 = 0.474, *p* value < 0.05), and a negative association (higher age at first birth, lower risk of long-term illness) among women in the Netherlands (OR, age 35+ = 0.313, *p* value < 0.05) and France (OR, age 35+ = 0.297, *p* value < 0.05). Among men, a U-shaped relationship was found in Switzerland (OR, age 20–24 = 0.067, *p* value < 0.05), while an inverted U-shaped relationship was found in Spain (OR, age 20–24 = 9.222 *p* value < 0.05). Age at last birth had a negative association with chronic conditions in Spain (OR, age 40+ = 0.405, *p* value < 0.05), a U-shaped association in Belgium (OR, age 25–29 = 0.61, *p* value < 0.05), and an inverted U-shaped association in Austria (OR, age 30–34 = 2.646, *p* value < 0.05) among women, while there was no significant relationship among men. Short birth intervals were associated with an increased risk of long-term illness among men in the Netherlands (OR 1.838, *p* value < 0.05). Long birth intervals were associated with a higher risk of long-term illness among women in Czech Republic (OR 1.799, *p* value < 0.05) and a lower risk among men in Belgium (OR 0.609, *p* value < 0.05). Experiencing the death of a child increased the risk of long-term illness among women in Germany and among men in the Netherlands.

**Table 3:** Long-term illness multivariate regressions—women

Long-term illness (yes vs. no)	AT	DE	SE	NL	ES	IT	FR	DK	CH	BE	CZ
# of Children (ref. = 0)											
1 Child	1.31	1.545	0.877	0.638	0.613	0.873	1.046	1.174	1.725	1.081	0.677
2 Children	1.482	1.393	0.912	0.618	0.759	0.821	1.246	0.968	1.353	1.023	0.986
3 Children	1.327	0.832	1.137	0.617	0.664	1.118	1.263	1.071	1.459	0.993	0.651
4 Children	1.787	1.462	1.42	0.565	0.608	1.197	1.306	0.763	1.185	1.084	1.133
5+ Children	5.592**	1.845	0.833	0.468*	1.032	0.949	2.733**	1.531	1.153	1.201	1.159
*N*	363	545	716	829	863	991	860	858	519	1087	658
Age at 1st birth (ref. < 20)											
20–24	0.88	0.742	0.520**	0.581	0.496	0.799	0.627	0.600*	1.142	0.916	0.965
25–29	0.913	0.807	0.467**	0.508*	0.465*	0.642	0.7	0.474**	1.024	0.88	0.645
30–34	1.244	0.603	0.455**	0.471*	0.461	1.084	0.935	0.736	0.411	0.794	0.598
35+	7.049*	3.701	0.822	0.313**	0.526	1.086	0.297**	0.72	1.872	0.504	0.654
Age at last birth (ref. < 25)											
25–29	1.762	1.285	0.732	0.789	0.946	0.794	0.676	0.968	0.605	0.610**	0.949
30–34	2.646**	1.294	0.648	0.643	0.61	1.15	0.707	0.957	0.513*	0.699	0.784
35–39	1.583	1.801	0.749	0.932	0.564	0.814	0.751	1.235	0.439*	0.695	1.638
40+	2.492	1.2	0.581	0.824	0.405**	1.071	0.677	1.415	0.8	0.537	1.725
*N*	332	497	663	744	787	908	781	792	438	973	637
Short birth interval (ref.: no)											
Yes	1.3	1.632	1.31	1.451*	1.255	0.923	0.881	1.102	0.755	1.095	0.84
Long birth interval (ref.: no)											
Yes	1.266	1.056	1.146	1.302	1.054	1.024	0.992	0.97	0.942	1	1.799**
*N*	263	397	572	667	715	738	623	686	375	782	532
Death of a child (ref.: no)											
Yes	0.824	2.454**	1.657	1.196	1.51	1.128	0.901	1.641	2.070*	1.114	0.769
*N*	332	497	663	744	787	908	781	792	438	973	637

Bold values reflect odd ratios that are significant at least at 5% level, i.e. *p* < 0.05

Odds ratios reported in the table; *p < 0.10, **p < 0.05, ***p < 0.01

**Table 4:** Long-term illness multivariate regressions—men

Long-term illness (yes vs. no)	AT	DE	SE	NL	ES	IT	FR	DK	CH	BE	CZ
# of Children (ref. = 0)											
1 Child	0.406	0.611	1.239	1.046	0.529	0.695	1.143	0.796	1.001	1.62	1.187
2 Children	0.31	0.978	1.184	0.905	0.559	0.697	0.733	0.774	0.633	1.234	0.848
3 Children	0.257	0.842	0.724	0.698	0.436*	0.699	0.91	0.986	0.783	1.314	0.867
4 Children	0.201*	0.994	1.669	0.65	0.578	0.578	0.998	0.584	1.955	1.488	1.073
5+ Children	0.313	0.431	1.166	1.379	0.641	0.722	1.397	1.345	0.112*	1.228	2.904
*N*	238	482	575	632	693	803	633	700	386	852	426
Age at 1st birth (ref. < 20)											
20–24	0.31	0.759	0.545	0.862	9.222**	0.266	0.941	3.648	0.067**	0.979	1.657
25–29	0.466	0.601	0.466	0.45	4.601*	0.261	0.922	3.368	0.080*	0.999	1.501
30–34	0.349	0.552	0.496	0.551	3.652	0.238	1.134	3.343	0.188	0.859	1.118
35+	0.249	0.34	0.407	0.773	4.188	0.313	0.873	1.852	0.082*	1.201	0.558
Age at last birth (ref. < 25)											
25–29	0.991	1.161	0.865	1.112	0.923	2.423	1.981	1.466	0.376	0.811	0.636
30–34	1.466	0.942	0.864	0.639	0.677	2.704	2.412*	1.744	0.557	0.887	1.267
35–39	0.941	0.566	0.688	1.265	0.585	2.591	1.844	1.511	0.582	0.736	0.47
40+	0.576	0.588	0.522	0.838	0.609	2.1	2.336	0.844	0.403	0.693	0.32
*N*	208	418	516	548	603	727	567	615	329	757	407
Short birth interval (ref.: no)											
Yes	1.332	1.139	1.202	1.838**	1.151	1.265	0.856	0.958	0.989	0.863	1.124
Long birth interval (ref.: no)											
Yes	0.935	1.047	0.834	1.337	0.973	0.908	1.277	1.460*	0.946	0.609**	1.156
*N*	166	324	458	490	545	589	483	544	291	615	344
Death of a child (ref.: no)											
Yes	1.241	2.17	0.847	3.635***	1.553	0.988	0.62	0.737	0.5	1.413	1.028
*N*	208	418	516	548	603	727	567	615	329	757	407

Bold values reflect odd ratios that are significant at least at 5% level, i.e. *p* < 0.05

Odds ratios reported in the table; *p < 0.10, **p < 0.05, ***p < 0.01

**Table 5:** Chronic diseases multivariate regressions—women

Chronic disease (yes vs. no)	AT	DE	SE	NL	ES	IT	FR	DK	CH	BE	CZ
# of Children (ref. = 0)											
1 Child	1.29	1.507	0.863	0.479*	0.84	0.641	1.241	1.331	1.75	1.376	0.703
2 Children	1.388	1.038	1.149	0.583	0.493	0.735	1.504	1.141	0.67	1.088	0.996
3 Children	0.696	0.612	1.238	0.796	0.371	0.743	1.744	0.921	1.249	1.042	0.828
4 Children	1.453	1.031	1.359	0.476*	0.604	0.792	1.226	1.099	0.767	0.844	1.232
5+ Children	1.3	0.856	1.408	0.637	0.363	1.434	2.639*	2.147	1.791	0.6	0.858
*N*	363	545	716	829	863	991	860	858	519	1087	658
Age at 1st birth (ref. < 20)											
20–24	0.433*	0.814	0.357**	0.596	0.629	1.102	0.357**	0.934	0.677	1.19	0.68
25–29	0.332*	0.85	0.291***	0.608	0.408	1.095	0.412*	0.709	0.773	1.256	0.464
30–34	1.241	0.635	0.408*	0.449	0.391	1.064	0.669	0.908	0.503	2.291	0.555
35+	0.638	1	0.59	0.330*	1	0.922	0.466	0.684	1.011	2.091	0.127**
Age at last birth (ref. < 25)											
25–29	1.207	1.575	0.865	1.338	1.428	0.972	1.029	1.293	0.628	1.181	0.896
30–34	1.204	1.045	0.669	1.477	1.012	1.134	0.934	0.955	0.58	1.612	0.654
35–39	0.903	1.153	0.632	1.248	1.334	0.782	0.947	0.897	0.407*	1.27	0.597
40+	1	0.513	0.537	1.59	1.333	0.631	1.886	0.99	0.388	1.514	1.152
*N*	332	497	663	744	787	908	781	792	438	973	637
Short birth interval (ref.: no)											
Yes	2.650*	1.34	0.948	0.899	1.164	0.845	0.895	1.173	0.942	1.165	0.567
Long birth interval (ref.: no)											
Yes	1.451	0.758	1.568	2.338**	1.464	1.162	1.032	1.147	0.829	0.646*	0.932
*N*	263	397	572	667	715	738	623	686	375	782	532
Death of a child (ref.: no)											
Yes	0.578	2.208	1.93	1.137	1.083	1.298	0.668	1.942	2.980**	1.216	1
*N*	332	497	663	744	787	908	781	792	438	973	637

Bold values reflect odd ratios that are significant at least at 5% level, i.e. *p* < 0.05

Odds ratios reported in the table; *p < 0.10, **p < 0.05, ***p < 0.01

**Table 6:** Chronic diseases multivariate regressions—men

Chronic disease (yes vs. no)	AT	DE	SE	NL	ES	IT	FR	DK	CH	BE	CZ
# of Children (ref. = 0)											
1 Child	0.315	0.59	2.014	0.686	1.184	0.968	1.107	0.98	0.682	0.895	0.319
2 Children	0.398	1.56	1.226	0.517*	1.225	1.299	0.67	0.781	0.410*	0.745	0.35
3 Children	0.319	0.915	0.706	0.503*	0.725	0.781	1.138	1.049	0.489	0.679	0.266
4 Children	0.249	0.607	1.032	0.468	1.58	0.997	0.831	0.977	0.91	0.913	1
5+ Children	0.454	0.425	4.227*	0.557	1.635	2.728	0.94	1.229	0.558	1.608	0.068
*N*	238	482	575	632	693	803	633	700	386	852	426
Age at 1st birth (ref. < 20)											
20–24	0.295	1.225	0.409	1.616	3.102	2.754	0.247	6.573**	1.529	8.336*	0.533
25–29	0.272	1.568	0.378	1.495	0.927	2.078	0.322	8.363**	1.979*	7.830*	0.518
30–34	0.377	1.227	0.459	1.359	0.891	2.218	0.321	5.650**	1.608	7.651*	0.695
35+	1	1	0.259	2.597	0.543	2.948	0.205	4.794*	1	7.309*	0.691
Age at last birth (ref. < 25)											
25–29	0.672	2.416	0.673	1.236	0.668	0.469	1.402	1.696	0.761	1.555	0.428
30–34	1.486	1.638	0.521	0.906	0.558	0.669	1.646	1.765	0.915	1.599	0.582
35–39	3.142	1.754	0.389	1.477	0.443	0.643	0.867	1.476	0.541	1.223	1.056
40+	0.931	1.145	0.319*	1.291	0.302	0.705	0.858	1.105	0.255	1.676	0.211
*N*	208	418	516	548	603	727	567	615	329	757	407
Short birth interval (ref.: no)											
Yes	0.653	1.013	0.787	1.277	1.608	0.585*	1.821	1.218	0.559*	0.569**	0.946
Long birth interval (ref.: no)											
Yes	4.095**	0.912	0.766	1.648	0.82	1.315	0.997	1.104	0.6	0.961	1.344
*N*	166	324	458	490	545	589	483	544	291	615	344
Death of a child (ref.: no)											
Yes	0.7	1.358	1.057	1.298	1.398	2.384	1.755	0.822	0.97	1.359	0.177**
*N*	208	418	516	548	603	727	567	615	329	757	407

Bold values reflect odd ratios that are significant at least at 5% level, i.e.* p* < 0.05

Odds ratios reported in the table; *p < 0.10, **p < 0.05, ***p < 0.01

Moving to Tables 5 and 6, the results for number of children and for age at last birth did not show any significant association with the risk of having a chronic disease, neither for women nor for men. There was a U-shaped association between age at first birth and the risk of being diagnosed with a chronic disease among Swedish (OR, age 20–24 = 0.357, *p* value < 0.05, OR, age 25–29 = 0.291, *p* value < 0.01) and French (OR, age 20–24 = 0.357, *p* value < 0.05) women, a negative association among Czech women (OR, age 35+ = 0.127, *p* value < 0.05), and an inverted U-shaped association among Danish men (OR, age 20–24 = 6.573, OR, age 25–29 = 8.363, OR, age 30–34 = 5.650, *p* value < 0.05). Also, there was no association between short birth intervals and chronic diseases for women, while short birth intervals decreased the risk of chronic diseases for men in Belgium (OR 0.569, *p* value < 0.05). Long birth intervals increased the risk of chronic diseases in the Netherlands for women and in Austria for men, while experiencing the death of a child was associated with a higher risk of chronic conditions among women in Switzerland and a lower risk among men in Czech Republic.

## Discussion

Analysing the relationship between fertility histories and chronic conditions later in life is necessary to understand the biosocial determinants of health and shed light on critical elements of the ageing process. This study extends the current body of literature by demonstrating the value of investigating cross-national differences in the association between fertility histories and the prevalence of chronic conditions, and to look at different measures of such chronic conditions. The results indicate that in European countries, age at first birth is more relevant to predict health outcomes than number of children, which has been more frequently used in the literature (see e.g. Dior et al. 2013; Engelman et al. 2010; Hardy et al. 2007). In most countries, women who experienced teenage pregnancy show a higher risk of developing chronic conditions later in life. This is likely due to the fact that young parents have fewer resources and social support to deal with the stress of parenthood and to cope with the economic strain of the situation (Falci et al. 2010). The negative consequences of teenage pregnancy can cumulate over the life course and result in worse health outcomes later in life, as hypothesized by the aforementioned life course models. The weaker association between parity and chronic conditions in older ages can also be explained by the fact that in European countries, the low variation in fertility rates might not be sufficient to detect effects that could be observed in populations with higher levels of fertility.

However, there are important gender differences, as results show that the number of children is more important for men and the death of a child is more important for women. Differences can also be observed across measures of health; for men, the number of children is significant only when looking at the prevalence of diagnosed chronic diseases, and age at first birth is significant only when looking at the number of chronic diseases. In addition, the association with chronic conditions exists only for some fertility characteristics, like parity and age at first birth, but for other variables—like length of birth intervals and age at last birth—the results are mixed and less clear.

Overall, the findings confirm the hypotheses introduced by several life course theories and by the health development model (Halfon and Hochstein 2002; Kuh and Shlomo 2004; Kuh et al. 2013). It is necessary to consider life course trajectories and their cumulative effects to understand health later in life and the ageing process. Moreover, findings show cross-country heterogeneity. In socio-democratic and continental countries, the association between fertility and chronic conditions—in particular between age at first birth and long-term illnesses—is statistically significant among women but not among men (except for Denmark), suggesting a stronger physiological link between fertility and health later in life. The fact that the association is significant only among women in these countries suggests an attenuation of negative social mechanisms related to parenthood (e.g. economic strain, stress), possibly through a more generous welfare system and more egalitarian gender norms. Given the less generous welfare regimes and lower gender equality in Southern and Eastern Europe, larger associations were expected in these countries; however, it was not the case. According to Esping-Andersen’s welfare regime theory (2013), welfare states have the power to influence individual behaviour and can be associated with different life course trajectories. In Scandinavian countries, where social benefits are large, life course trajectories tend to be more heterogeneous, fertility rates higher, gender equality fostered by social and childcare policies, and the national health care system can reduce health inequality at older ages. Southern and Eastern European countries, on the contrary, are characterized by low levels of welfare provisions and a strong reliance on the family as a locus of support (Trifiletti 1999). These countries tend to have lower fertility rates, a higher mean age at childbearing, stronger gender norms, and greater levels of intergenerational transfers that can influence social support and health outcomes in older ages. The results of the present study cannot be fully explained through this theoretical model or by the national differences in gender equality at the institutional and household levels (McDonald 2000a), as some of the cross-national differences contradict the hypothesized outcomes. It is important to note that the low significance of some of the associations found in the analyses may be due to small sample sizes. More research is needed to explain cross-national findings, possibly using larger samples.

Population ageing is one of the most important demographic phenomena taking place in developed countries. This study shows that it is important to consider life course events, such as childbearing, in order to understand health outcomes at older ages and to investigate the cumulative effects that can lead to healthier or unhealthier ageing processes.

This study is not without limitations. The sample size for each country is small, especially when the analysis was stratified by gender and when the age at childbearing, birth intervals, and death of a child were analysed. Therefore, the power of the analysis might be limited. Moreover, the measure of chronic disease as diagnosed by a doctor is only partially objective, as it reflects such diagnoses through self-reports; this measure would be more reliable if a doctor or a nurse reported the response. That might be why the results do not show large differences in the relationship between fertility and chronic diseases across the two measures. However, it is important to compare different measures for chronic conditions, given the fact that the previous literature has found mixed findings depending on the measure used. Finally, as not all European countries were included in the sample the results cannot be generalized to Europe as a whole without some caution.

Future research should focus more on cross-national comparisons because there are differences across countries that cannot be entirely accounted for by welfare system theory, possibly looking at historical and cultural explanations. Additionally, different measures of health should be used, comparing self-reported measures with objective measures such as biomarkers and diagnoses based on health examinations performed by doctors. Moreover, given the interconnectedness of the fertility aspects considered here, it would be useful to examine fertility trajectories as a whole, while also taking partnership histories into account.

## Conclusions

The results of the present study are useful to identify groups in the population that are more at risk of developing chronic conditions and unhealthy ageing. In particular, findings show the importance of age at first birth among women; having a teenage pregnancy or having the first child after age 35 is associated with an increased risk of chronic conditions later in life. These recognized groups may benefit from tailored interventions or more health monitoring, in order to prevent future health complications. This is increasingly important, given current trends of fertility decline and increase in the age at childbearing, and given the phenomenon of population ageing that is taking place across Europe.

## Footnotes

1 SHARE data are anonymized, and informed consent was obtained from all individual participants included in the survey.

2 For more details, see Chapter 8 “Fieldwork monitoring and survey participation 101 in the fifth wave of SHARE” in *Malter and Börsch-Supan* (*Eds.*) (2015)*. SHARE Wave 5: Innovations & Methodology. Munich: MEA, Max Planck Institute for Social Law and Social Policy*.

3 These are the abbreviations used for the countries in the sample: AT = Austria, DE = Germany, SE = Sweden, NL = the Netherlands, ES = Spain, IT = Italy, FR = France, DK = Denmark, CH = Switzerland, BE = Belgium, CZ = Czech Republic.

4 The list of conditions on the card showed to the respondents includes the following: a heart attack including myocardial infarction or coronary thrombosis or any other heart problem including congestive heart failure; high blood pressure or hypertension; high blood cholesterol; a stroke or cerebral vascular disease; diabetes or high blood sugar; chronic lung disease such as chronic bronchitis or emphysema; cancer or malignant tumour, including leukaemia or lymphoma, but excluding minor skin cancers; stomach or duodenal ulcer, peptic ulcer; Parkinson disease; cataracts; hip fracture; other fractures; Alzheimer’s disease, dementia, organic brain syndrome, senility or any other serious memory impairment; other affective or emotional disorders, including anxiety, nervous or psychiatric problems; rheumatoid arthritis; osteoarthritis, or other rheumatism; none; other conditions, not yet mentioned.

5 No education, primary or lower secondary, upper secondary, and tertiary education according to the ISCED scale.

6 Single, married or cohabiting, divorced or separated, and widow (at wave 5).

7 Parents’ education (highest between mother and father), if living with both biological parents when 10, the number of people per bedroom when 10, books in the household, housing conditions.

8 Self-reported health, had vaccines, had a health care source, missing school for a month or more due to health problems, being hospitalized for a month or more.

9 Descriptive statistics are weighted using calibrated weights to take into account attrition, non-response rate, and sample differences across countries.

10 Given the large number of regression models in the analysis (due to different outcomes and different fertility measures), no bivariate associations are reported in “Results” section, but are available upon request.
